# A network of change: united action on research integrity

**DOI:** 10.1186/s13104-022-06026-y

**Published:** 2022-04-14

**Authors:** Thomas Rhys Evans, Madeleine Pownall, Elizabeth Collins, Emma L. Henderson, Jade S. Pickering, Aoife O’Mahony, Mirela Zaneva, Matt Jaquiery, Tsvetomira Dumbalska

**Affiliations:** 1 School of Human Sciences, University of Greenwich, London, England; 2 Institute for Lifecourse Development, University of Greenwich, London, England; 3 University of Leeds, Leeds, England; 4 University of Stirling, Stirling, Scotland; 5 University of Surrey, Surrey, England; 6 University of Manchester, Manchester, England; 7 School of Psychology, Cardiff University, Cardiff, Wales; 8 Oxford University, Oxford, England

**Keywords:** Research integrity, Reproducibility, Research stakeholders, Collaboration, Registered Reports, Open Data, Open research

## Abstract

The last decade has seen renewed concern within the scientific community over the reproducibility and transparency of research findings. This paper outlines some of the various responsibilities of stakeholders in addressing the systemic issues that contribute to this concern. In particular, this paper asserts that a united, joined-up approach is needed, in which all stakeholders, including researchers, universities, funders, publishers, and governments, work together to set standards of research integrity and engender scientific progress and innovation. Using two developments as examples: the adoption of Registered Reports as a discrete initiative, and the use of open data as an ongoing norm change, we discuss the importance of collaboration across stakeholders.

## Introduction

Evidence of a number of problematic practices and norms across the research cycle give us good reason to doubt the credibility of much research [12, 15]. This, coupled with mostly unsuccessful attempts to replicate core research findings in psychology [18] and elsewhere [5], exemplifies the far-reaching issues of research integrity that the scientific community currently face. Researchers prioritising research transparency, quality, and culture have driven changes in research norms across the world, with open science/scholarship initiatives playing a central role in developing and championing new approaches and standards.

Whilst the scale of change achieved in the last decade is notable, a central barrier to sustainable change in integrity norms is the extent to which all research stakeholders collaborate to embed and progress such developments [19]. Here, we summarise two developments, open data and Registered Reports, which can tackle this wider crisis of science through increased transparency, research quality, and changes to research culture. We discuss how the research community needs to collectively tackle such issues, acknowledging how action from one stakeholder can alter demands and value for other stakeholders, thus requiring coordinated action.

## Main text

### Open Data

One driver of the current crisis is a lack of transparency—a lack of open sharing of data and materials. As observed during the COVID-19 pandemic, making data openly accessible is transformative for scientific and public understanding, providing accountability within psychological research [1]. Unfortunately, sharing data has been uncommon historically, and when materials and data are not shared, researchers, funders, and journals cannot adequately assess the robustness of published work, slowing scientific progress. Openness is also an important facilitator of reproducibility, as researchers often struggle to reproduce analyses or conclusions without access to associated datasets (e.g., Wicherts et al., 2016).

Inaccessibility of data, and thus low transparency, makes attempts to progressively build upon previous research inefficient for funding and researcher hours. It is harder to replicate and establish the boundaries of effects and to evaluate the quality of work. It can also hinder error detection and correction, and the identification of fraud (e.g., [22]. Therefore, research transparency can have multifaceted direct and indirect consequences on the quality and speed of research developments, and should be a priority for stakeholders.

Advocating for transparency in research requires a *cultural* shift and a fundamental realignment of expectations. Currently, scientific norms encourage researchers to state that data is available “upon reasonable request”, but subsequent rates of data sharing by request are unacceptably low [13],Wicherts et al., 2016; [6]. A priority for the scientific community should be ensuring that data are safely preserved, conform to the FAIR principles (Findable, Accessible, Interoperable, Reusable [23], and are openly available for re-use and re-analysis where possible. Table 1 explores the interconnected demands placed upon all stakeholders of research regarding open data.

**Table 1:** Interconnected Roles for Stakeholders in Open data and RRs

	Individual researchers	Research support	Institutions (universities)	Funders	Publishers	Government bodies
Roles for Open Data	Collect and/or curate research data	Resource infrastructure enabling data storage and sharing	Prioritise and fund training about transparency and the infrastructure offered by research support for sharing research materials	Establish policies regarding the level of transparency and openness required for funding	Maintain and enforce author guidelines that specify how research data/materials are stored and shared as a condition of publication	Provide/signpost recommendations, support, and structures for all stakeholders (e.g., templates, training)
	Manage and deposit data using an appropriate storage location	Make financial choices about journal subscriptions and partnerships	Acknowledge openness as part of research quality evaluations during appraisals	Evaluate adherence to transparency policies and communicate consequences for non-compliance		Audit institutions, funders and publishers
						Facilitate collaborations across stakeholder groups
						Facilitate, communicate, and champion development of transparency norms and practices
Roles for RRs	Plan, develop, conduct, and disseminate research findings	Offer training that enables researchers to make educated and strategic choices about publishing	Prioritise and fund training which supports researchers to prioritise higher quality evidence and more transparent and rigorous research processes	Prioritise the role of rigour and transparency explicitly when assessing the quality of work being considered for funding	Assess research quality for publication based on journal criteria	
	Choose publication and feedback workflow (e.g., RR, traditional, etc.)		Incentivise and appraise staff on subsequent transparency and rigour in research practices		Capture and evaluate meta-data to identify meaningful trends and development areas	

Researchers that are willing to share their data face challenges in resourcing and knowing how to do so ethically whilst conforming to FAIR principles [23]. To facilitate data sharing, co-ordinated change is needed across stakeholders. For example, changes to journal data availability statement policies can facilitate sharing practices (e.g., [10], but this increases demands upon training, support and infrastructure of consequence to researchers, research support (e.g., libraries, technicians), universities, and funders [11]. Table 2 considers the various responsibilities each research stakeholder have towards co-ordinated reform of standards.

**Table 2:** Interconnected Recommendations for Stakeholders in open data and RRs

	Individual researchers	Research support	Institutions (universities)	Funders	Publishers	Government bodies
Shared Recommendations	Sign and follow the principles of DORA, such that research is evaluated on its own merits, with transparency as a valued dimension, rather than the journal/place of publication.					Undertake rigorous and systematic evaluations of research environments to ensure sufficient structure and support within and across stakeholder groups. Priority should be given to ensuring cohesiveness between actions from the different stakeholder types, identifying and sharing best practices, and identifying specific groups or institutions in need of more localised interventions.
Open Data Specific Recommendations	Incorporate open practices (as appropriate) throughout the research workflow.	Invest in infrastructure for sustainable approaches to data management e.g., automated data archiving (see [20]).	Hire meta-scientists to improve and encourage open data norms.	Mandate data sharing statements, and conduct regular audits to ensure adherence and quality.	Mandate open data (with appropriate caveats where not possible e.g., partial data, embargoed, other gatekeeping etc) and FAIR principles (e.g. meta-data and codebooks).	Encourage and signpost infrastructures available to connect researchers/ institutions and improve research quality.
	Use positions of power (e.g., line managers, project leads) to communicate expectations, share good practice, and provide practical support for improving transparency.	Offer training regarding best practices in transparency.	Promote transparent scientific practices in hiring and promotion decisions and awards (e.g., recognising preregistrations, RRs and pre-prints).	Recognise transparency track record as a positive characteristic when assessing applications.	Prioritise policy and structural developments in accordance with TOP guidelines [17].	Support and champion development and evaluation of new initiatives like RRs.
		Responsibly use funding to prioritise partnerships with organisations committed to transparency e.g., data repositories and open access journals.	Instigate curriculum changes so all students have an understanding and experience of open practices.			Audit adoption of RRs and similar initiatives and compile an evidence-base which evaluates the implications of wider adoption.
Registered Report Specific Recommendations	Where appropriate, submit research using the Registered Report format or create a time-stamped preregistration.	Ensure adequate training is available to researchers in research design, analysis, and research integrity.	Realign incentive structures to value quality and integrity over quantity or metrics. E.g., value use of RRs when appraising academic staff.	Funding assessment criteria should prioritise the importance of research question, quality of method, and transparency.	Journals/ publishers should consider adopting RRs (amongst other innovations) and provide clear author guidelines (templates: osf.io/pukzy/).	
	Engage in methods, statistics, and open practices training.	Go further, e.g., subject librarians can assist in projects or trained statisticians can verify code. Research support can be the provision of an environment that promotes collaboration.	Publicly declare the disconnect between journal impact factor and research quality (e.g,[9]) and make associated changes to structures and processes.	Explore RR Funding Partnerships, or similar initiatives, to encourage simultaneous funding and publication of research.	Publication should be offered on the transparency, quality of research question and methodology; not on novelty or positive results. Policies relating to such should be implemented and audited.	
	Those in positions of power should role model use of RRs (and similar) as responsible and sustainable publication practices, encouraging their teams/students to do the same.				For confirmatory work, require preregistration with a concrete theoretical background and specific falsifiable hypotheses.	

### Registered Reports

Research quality is a vital component of research integrity. We cannot promote better integrity of research if we do not first consider how the quality (i.e., robustness, reliability, and validity) can be improved. One barrier to research quality actively propagated by many publishers and journals is ‘publication bias’, whereby null/non-significant results are much less likely to be published than statistically significant findings. This incentivises questionable practices such as p-hacking data to ‘find’ a significant result, or selectively reporting significant results [2, 8]. This directly contributes to the crisis because it makes publication contingent upon the results of the work, rather than the theoretical significance and methodological rigour of the research.

**Fig. 1:**
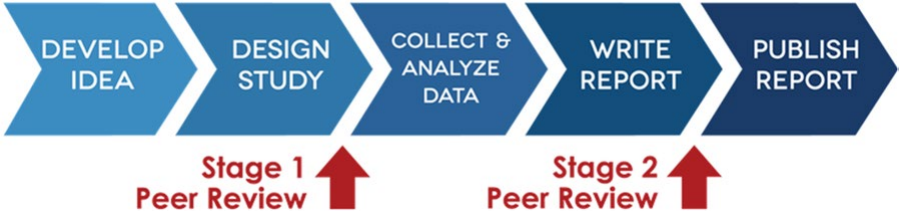
The RR Publication Pathway (image from Centre for Open Science)

Concerned by publication bias, researchers have developed several initiatives to improve research practices and standards in methodology and publishing. Deviating from the traditional publication route where papers are peer-reviewed following study completion, Registered Reports (RRs) are one such innovation in publication. At Stage 1, the introduction, hypotheses/research questions, methods, and analyses undergo peer-review before data collection. This feedback can identify flaws in the protocol and allows substantive changes to be made before using resources (e.g., funding, participant time). Work receives in-principle acceptance from the journal, whereby the subsequent completed (Stage 2) report will be published regardless of the findings, if the authors have collected and reported data according to Stage 1 [3]. RRs reduce publication bias because acceptance is based on the importance of the research question and methodological rigour, rather than the results. This reduces pressure to produce significant results and counters the incentives that drive selective reporting and other questionable research practices [4]. RRs are valuable amid ongoing concerns of widespread ‘false-positive findings’ in the published literature, as hypotheses are supported much less frequently among RRs than conventional research articles [21], providing initial evidence for the value of the approach (Fig. 1).

Further structural support is needed in order to implement RRs more widely, including training, funding, and wider journal adoption. See Tables 1 and 2 outlining the interconnected roles and responsibilities of research stakeholders for RRs. Registered Report Funding Partnerships have been proposed as a method of extending the RR model by integrating it with the grant funding process, such that researchers receive both funding and in-principle acceptance for publication based on the integrity of the theory and methods. Combining funding and publication decisions may streamline processes and reduce the burden on reviewers, while also providing the aforementioned benefits of RRs in reducing questionable research practices and publication bias [14]. Such RR-funding partnerships, and similar innovations for drug marketing authorisation [16], offer important and innovative examples of how stakeholders and processes can be unified to improve standards for research quality.

### Outlook

Overcoming the issues underlying the current crisis requires united action across research stakeholders. For example, individuals may wish to conduct RRs, but journals must offer this option and funders must value and incentivise such work. Similarly, journals can mandate open data sharing, but researchers require training, support and infrastructure to facilitate this. Initiatives designed to improve research integrity should be mapped out with consideration to the different demands and value provided to each of the different stakeholder groups. This allows obstacles to be anticipated and encourages co-ordinated action, increasing the likelihood of such initiatives becoming sustainable.

Acknowledging our priorities of transparency, rigour and culture, open data and RRs represent only two initiatives which require more collective action. While we focused here on open data, transparency could also be prioritised by promoting open sharing of research materials, which rely on the same mechanisms. Similarly, we focused on RRs as one method to alleviate publication bias, but other initiatives, such as open peer review and crowd-sourced open review, also represent promising avenues to improve research integrity. Thus, the priorities and ideas here should be viewed as a starting point for a wider, more comprehensive consideration of how the transparency, quality, and culture of research, and thus integrity, can be improved *together*.

## Data Availability

Not applicable.

## Abbreviations

DORA: Declaration on Research Assessment
FAIR: Findable, Accessible, Interoperable, Reusable
RRs: Registered Reports
